# Overwintering under ice: A novel observation for an Australian freshwater turtle

**DOI:** 10.1002/ece3.11578

**Published:** 2024-07-14

**Authors:** James Dowling, Deborah S. Bower, Eric J. Nordberg

**Affiliations:** ^1^ School of Environmental and Rural Science University of New England Armidale New South Wales Australia

**Keywords:** brumation, *Chelodina longicollis*, eastern long‐necked turtle, freshwater, ice, overwintering

## Abstract

Frozen water bodies provide a physiological challenge to fauna by physically limiting access to atmospheric oxygen. To tolerate low temperatures, reptiles use brumation as a physiological strategy in winter. Cryptodira vary in their tolerance to freezing conditions but the extent of tolerance in pleurodirans is largely unknown. Australia's freshwater turtles inhabit warmer regions with less severe winters and have well‐developed mechanisms to cope with high temperatures and drying waterbodies, rather than extreme cold tolerance. *Chelodina longicollis* is a widespread Australian freshwater turtle species that tolerates high temperatures and desiccation during hot, dry periods while also undergoing brumation during winter months. Despite extensive research, limited observations exist on their behaviour during severe winter periods at the extremes of their range. In an 11‐month tracking study, we monitored adult *C. longicollis*, noting their movements, locations, and temperature weekly. We observed an adult female *C. longicollis* which, during a seven‐month period within a single creek pool, survived brumation in extreme cold water including a 15‐day period of total freezing of the surface water. After the ice melted following a rain event, the turtle was recaptured alive. This marks the first observation of brumation for an Australian chelid species under ice.

## Graphical abstract

An Australian freshwater turtle, *Chelodina longicollis*, was observed undergoing brumation under the ice of a frozen creek pool for a period of at least 15 days, marking the first such observation for this species. This novel finding suggests potential cold tolerance in pleurodiran turtles and highlights the importance of understanding thermal tolerance in the face of changing climates.

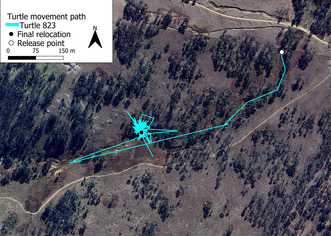

## 1 OBSERVATION

Cold environmental conditions reduce the activity and metabolism of many species in temperate regions (Geiser, 2020; Hut et al., 2002; Nordberg & Cobb, 2016, 2017; Staples, 2016; Taylor & Nol, 1989; Tøien et al., 2011; Turbill & Geiser, 2008). As lower temperatures reduce the physiological and behavioural performance of ectotherms (Huey & Stevenson, 1979; Taylor et al., 2021), individuals may brumate until the surrounding environment warms, and normal function can resume (DeGregorio et al., 2017; Grobman, 1990). For preferentially aquatic species, such as freshwater turtles, overwintering in water may offer the added advantage of thermal buffering from freezing surface temperatures (Taylor & Nol, 1989) and refuge from predation during periods of inactivity (Greaves & Litzgus, 2007). However, aquatic environments may pose challenges for overwintering turtles due to water surfaces freezing and preventing the exchange of atmospheric oxygen with the water (St. Clair & Gregory, 1990). Additionally, dissolved oxygen decreases under ice, and animals may encounter anoxic sediments when burying as a mechanism to buffer against cold temperatures (Brown & Brooks, 1994; Jackson & Ultsch, 2010).

While the winter activities of North American freshwater turtles are well documented (Robichaud et al., 2023), observations of Australian chelids have largely been limited to areas where winters are relatively mild (Seebacher et al., 2004, Van Dyke et al., 2023) or in laboratory settings (Chessman, 2019). Freshwater turtles are widespread along coastal Australia, with 25 native freshwater species across the continent (Van Dyke et al., 2018). The eastern long‐necked turtle, *Chelodina longicollis*, has an extensive distribution, ranging from the Fitzroy River in Northern Queensland to Port Lincoln, South Australia, at its most southwestern extent (Kennett et al., 2009). Introduced populations have become established in the cold climates of Tasmania and on islands of the Bass Strait, largely due to the release of domestic pets (Fearn, 2013; Ferronato et al., 2015). Despite the potential for eastern long‐necked turtles to endure icy winters, little is known of the brumation activity of adult *C. longicollis* outside of brumating both terrestrially and underwater (Kennett et al., 2009). Here, we describe the brumation of an adult *C. longicollis* female in a pool with a continually frozen water surface for a period of at least 15 days. Our observations were made during a study of the spatial ecology of *C. longicollis* in the New England area of New South Wales (NSW), Australia. The New England Region of NSW likely represents the upper elevation limit of *C. longicollis* (Kennett et al., 2009), with populations recorded at 1340 m (−30.4014, 151.6289; D.S. Bower & E.J. Nordberg unpublished data). Turtles in this study were captured from Duval and Sandy Creeks on the University of New England's Newholme SMART farm from 9 October 2022 to 4 December 2022. These turtles were fitted with GPS data loggers (Advanced Telemetry Systems W510 Wildlink) and released at the point of capture. Data loggers recorded ambient temperature, percentage of time spent moving in the 15 min prior to logging via accelerometer, elevation, and GPS location if out of water and the logger could connect to satellites.

**FIGURE 1:**
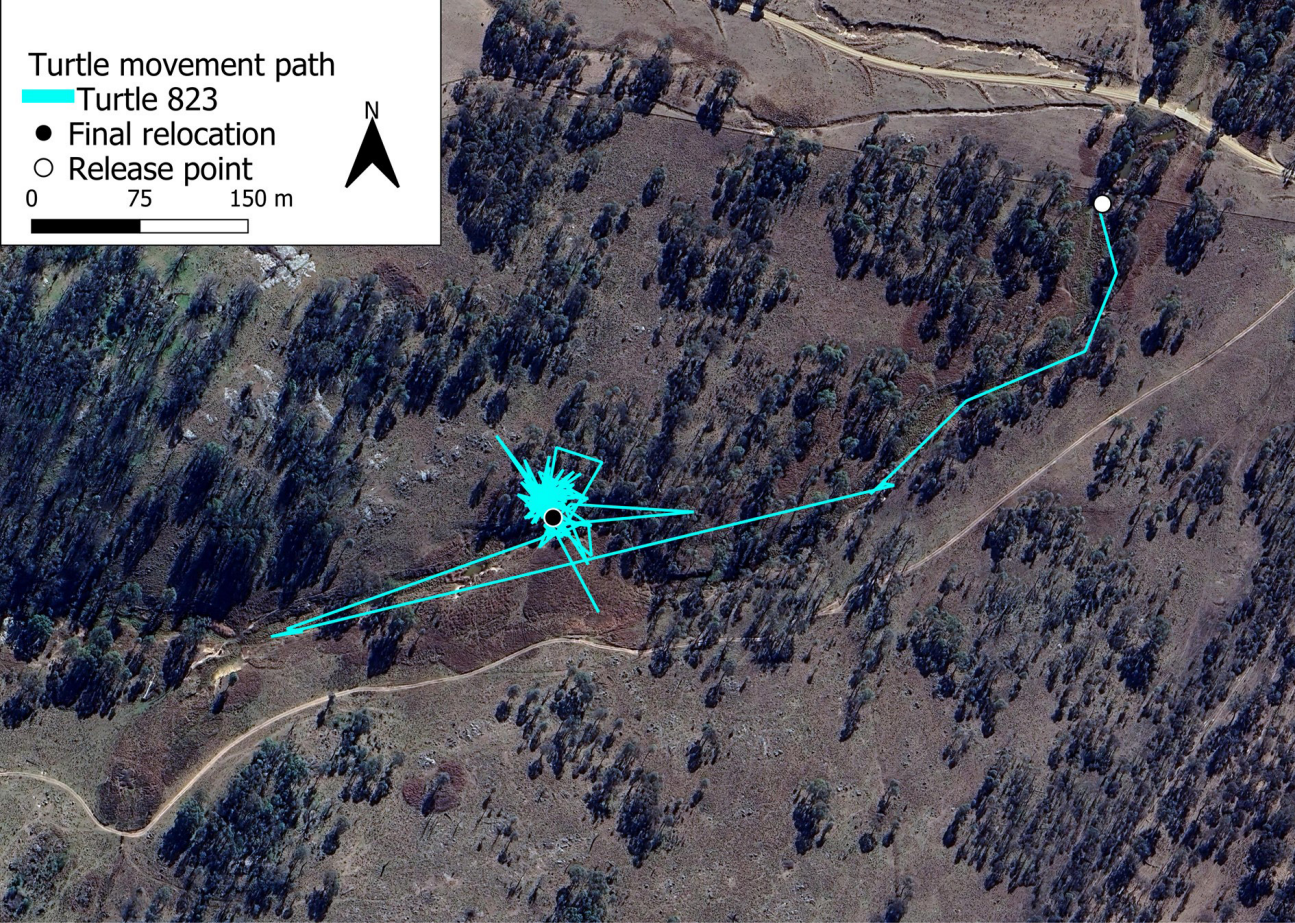
The turtle's movement path, reconstructed through GPS fixes on the attached data logger and weekly relocations, largely followed the creek line of Sandy Creek from the release point (white circle) to its last relocation point (black circle). The final relocation point falls within the creek pool in which the turtle overwintered, and the cluster of movement surrounding this point encompasses the movement within the pool itself and the area in which the turtle aestivated.

Following its release on 19 November 2022, a *C. longicollis* moved 590 m south‐west from its original point of capture and release, Sandy Creek (−30.4248, 151.6511), to a small, shallow pool (−30.4265, 151.6482) in an ephemeral creek line (Figure 1). This creek pool measured 11 m^2^ and had a depth that fluctuated between 0.3 and 0.5 m throughout the period of observation. The creek pool was located at the bottom of a highly eroded gully and was approximately 2.5 m below the average ground level of the surrounding countryside. To the north, a moderately wooded hillside shaded the pool from direct sunlight for most of the day during winter months. Additionally, Broadleaf Cumbungi (*Typha orientalis* C. Presl) grew thickly throughout the pool and provided significant shade from direct sunlight. The turtle displayed high site fidelity, spending a total of 7 months (7 February 2023–28 September 2023) in the vicinity of this pool. During this time, the turtle aestivated for 5 weeks (28 March 2023–2 May 2023) in thick grass directly adjacent to a fallen log, 35 m north of the pool. Following this brief period of aestivation, the turtle returned to the water where it was relocated the following week (9 May 2023) and stayed for the rest of the study.

**FIGURE 2:**
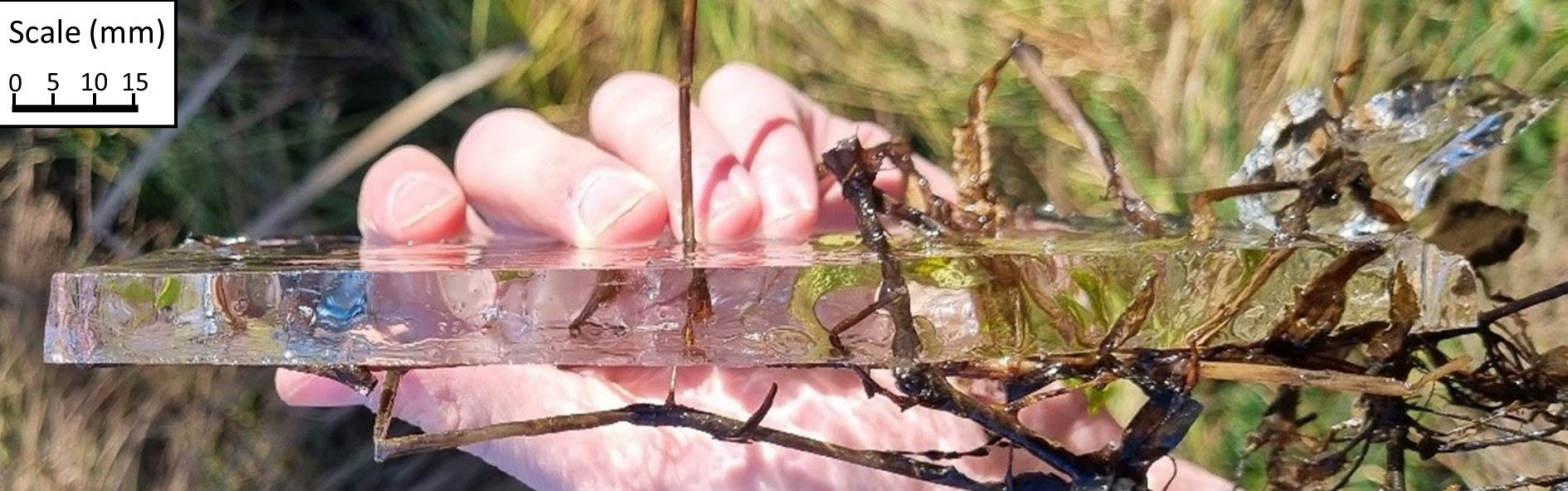
Surface ice thickness on 9th June 2023, recovered from within 2 m of the turtle's location. Ice at the edges of the water body exceeded this thickness by 10 mm in following weeks.

**FIGURE 3:**
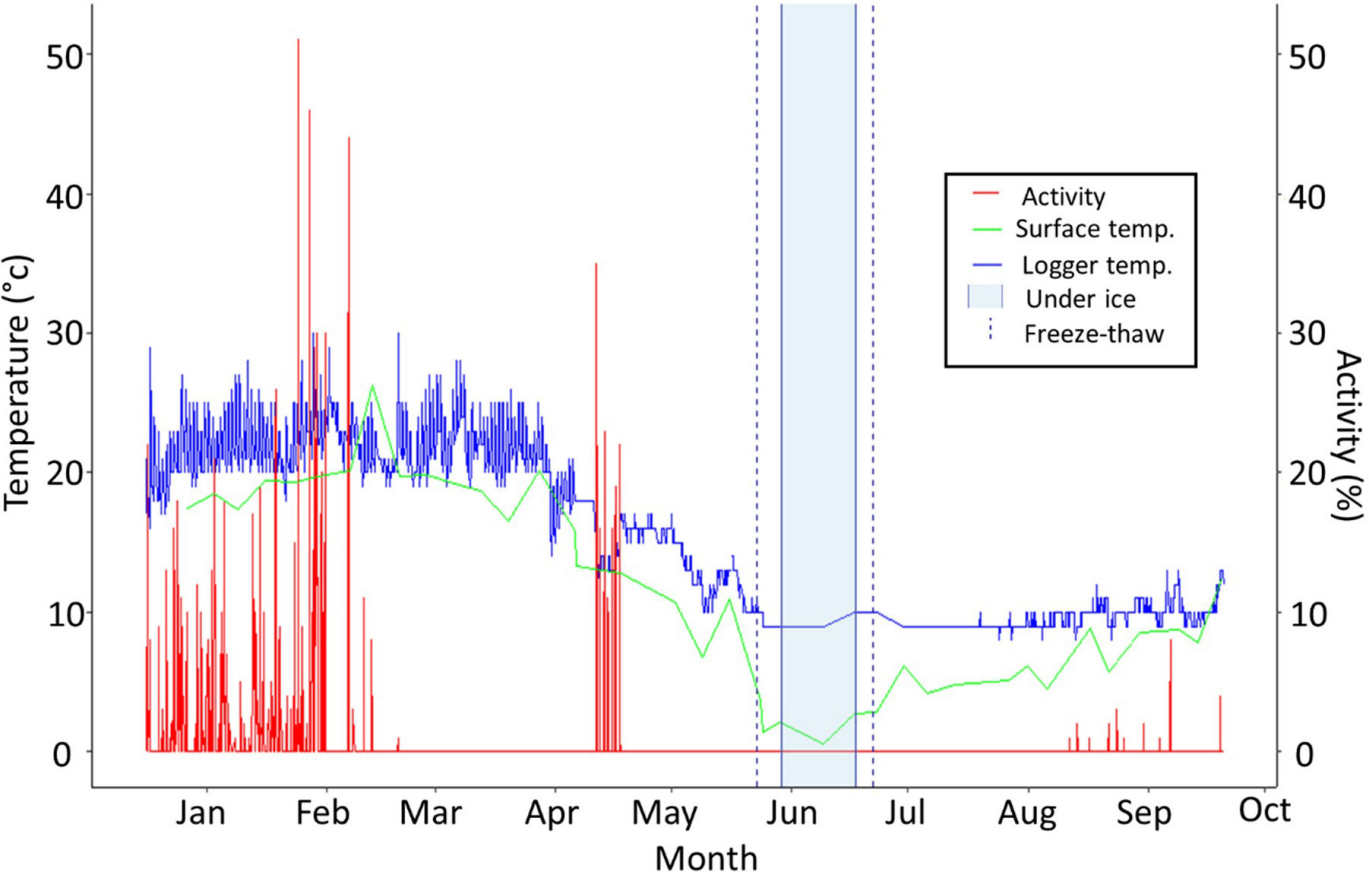
Recorded temperatures measured at the water's surface (green) and downloaded from the data logger (blue) throughout the tracking period for turtle 823. Activity (red) represents the proportion of time (%) that the data logger accelerometer registered movement in the 15 min prior to data logging. The period of brumation beneath the ice (light blue) is shown in addition to the observations in which the creek pool routinely froze and melted daily.

We first observed the surface water of the pool had frozen on 23 May 2023. However, at the time, higher temperatures melted the surface ice daily. The pool's surface froze on 29 May 2023, with a considerably thicker ice layer (approximately 8 mm). The pool remained frozen between 5 and 9 June 2023, with significantly thicker ice than in surrounding pools which received direct sunlight and were located outside of deep gullies. The thickness of ice in the pool in which the turtle was located (>15 mm) suggested it had been frozen for an extended period compared to the other waterbodies (Figure 2). On 17 June 2023, the ice reached its thickest depth, estimated to be 25–30 mm. During this time, surface water temperatures beneath the ice (taken from the top 5 cm of water using Cason ABS professional thermometer) ranged from 0.2 to 2.1°C. Shaded air temperatures (measured 1 m above ground level) ranged from 8.3 to 11.4°C when measured during observation periods at 10–11 am (Figure 3).

Rainfall on 22 June 2023 increased the water temperatures (6.7°C) and melted the ice that had previously covered the creek pool. The creek pool refroze on 30 June 2023, when water temperature fell to 2.8°C; however, during that time, the ice thawed daily.

We located and recovered the turtle on 10 July 2023. It was located at a depth of 0.3 m and was partially buried in the sediment of the creek bed. The turtle was alive despite experiencing extreme drops in water temperature. Data downloaded from the attached GPS data logger recorded water/substrate temperatures ranging from 8 to 12°C throughout the period in which the creek pool had been continuously under ice (Figure 3). This may reflect the sediment temperatures, in which the turtle was partly buried, providing an insulative buffer from the colder surface water temperatures (Taylor & Nol, 1989). After this observation, we validated our logger temperature measures against our handheld thermometer at a range of temperatures including an ice bath. The logger and handheld thermometer returned temperatures within 0.5°C of each other.

We observed four additional instances of turtles in water bodies where we measured surface water temperatures below 2°C throughout the study period. The environmental conditions of the locations in which turtles brumated were generally similar; creek pools with water depths between 0.4 and 0.7 m and dominated by reeds with surrounding surface vegetation exceeding 50%. While the surface of these creek pools did not appear to freeze completely, water at pool edges froze for brief periods.

To our knowledge, this is the first record of a pleurodiran turtle overwintering under ice for an extended period. Reports of aquatic brumation/hibernation in Australian freshwater turtles have been limited to two species which use bimodal respiration to survive extended submersions (Fielder, 2012; Gordos et al., 2003). While our observation demonstrates the ability of *C. longicollis* to survive low water temperatures, further research would be useful to unravel the extent of cold tolerance in pleurodirans and how this compares to the broad tolerance of Cryptodira, which may be ancestral to turtles generally (Packard et al., 1999). Further investigation is also warranted to assess the extent of vertical temperature stratification under ice and how partial burial in sediment affects turtle survival during severe winters. In the face of a changing climate, understanding species' thermal tolerance range is critical for predicting responses to not only increasing temperatures but also more intense and frequent extreme weather events, including frosts and cold snaps (Zhang et al., 2016). Additional experimental trials of the extent of cold tolerance of *Chelodina longicollis* would enable a greater mechanistic understanding of their physiological limits.

## AUTHOR CONTRIBUTIONS

**James Dowling:** Data curation (lead); formal analysis (lead); investigation (lead); methodology (equal); project administration (equal); writing – original draft (lead). **Deborah S. Bower:** Conceptualization (lead); project administration (equal); supervision (equal); validation (equal); visualization (equal); writing – review and editing (equal). **Eric J. Nordberg:** Conceptualization (equal); project administration (equal); supervision (lead); validation (equal); visualization (equal); writing – review and editing (equal).

## CONFLICT OF INTEREST STATEMENT

The authors declare that they have no known competing financial interests or personal relationships that could have appeared to influence the work reported in this paper.

### OPEN RESEARCH BADGES

This article has earned an Open Data badge for making publicly available the digitally‐shareable data necessary to reproduce the reported results. The data is available at 10.5061/dryad.stqjq2c9q.

## Data Availability

The data on which the analyses were conducted have been deposited in DRYAD: DOI: 10.5061/dryad.stqjq2c9q.
